# Supplementary material for: Inter-subject phase synchronization differentiates neural networks underlying physical pain empathy
Source: Soc Cogn Affect Neurosci. 2020 Mar 3;15(2):225–33. doi: 10.1093/scan/nsaa025 (PMC7304508; doi:10.1093/scan/nsaa025)
Supplement: scan-19-373-File005_nsaa025 [file scan-19-373-file005_nsaa025.docx]

**Supplementary Material**

Xu et al.,

**Assessment of number of independent components and replication**

We compared 10 and 15 component ICA solutions in the current data set. All participants were randomly split into two subgroups (original sample and confirmatory sample) three times for replication and assessment of number of components. ‘Replicability’ was measured in three ways: 1) the number of components that had ‘matching’ component pairs in the confirmatory sample in terms of visual examination between the original and confirmatory ICA solutions, 2) the spatial correlation between the ‘matching’ component pairs from the original and confirmatory ICA solutions, and 3) the temporal correlation between the ‘matched’ components from the original and confirmatory ICA solutions. This replicability analysis also allowed an assessment of the degree to which the synchronization approach replicates across samples. Results suggested that the 10-component ICA solution (1st/2nd/3rd time of grouping: matching components: 9/9/9 out of 10; rSpatial: 0.69/0.66/0.64, rTemporal: 0.73/0.70/0.69) was more replicable compared with the 15 component ICA solution (matching components: 13/13/13 out of 15; rSpatial: 0.54/0.61/0.52, rTemporal: 0.65/0.67/0.63). Thus, results from the 10-component ICA solution are presented.

**Fig. S1** Unclassified component in pain empathy task. C10 was labeled as unclassified based on voxel weight spatial patterns in white matter and cerebrospinal fluid regions. This component also could not be replicated in the split half sample replication.


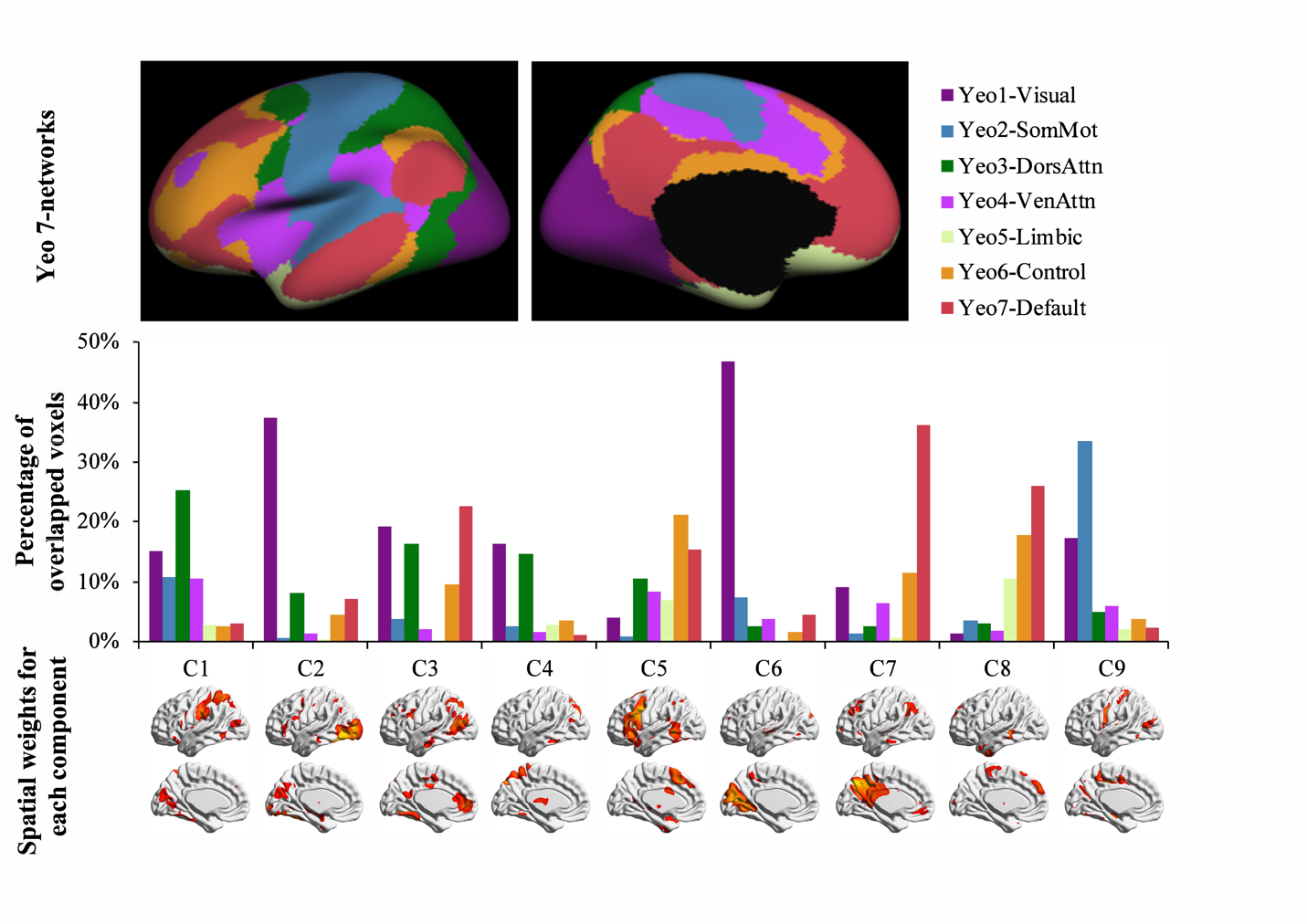


**Fig. S2 Overlap with 7 Yeo Networks for each component.**

**Table S1** Temporal correlation between ICA components and GLM hypothesized reference functions.

|  | Physical  Pain | Physical  Control |  | Affective  Pain | Affective  Control |  |  | Physical | Affective |  | Pain | Control |
| --- | --- | --- | --- | --- | --- | --- | --- | --- | --- | --- | --- | --- |
| C1 | 0.55* | 0.15 | † | -0.37* | -0.32* |  |  | 0.57* | -0.56* |  | 0.15 | -0.14 |
| C2 | 0.25* | 0.21 |  | -0.57* | -0.36* | † |  | 0.38* | -0.75* |  | -0.25* | -0.12 |
| C3 | 0.42* | 0.32* |  | -0.42* | -0.48* |  |  | 0.60* | -0.73* |  | -0.00 | -0.13 |
| C4 | -0.20 | -0.31* |  | 0.40* | 0.26* |  |  | -0.41* | 0.53* |  | 0.16 | -0.04 |
| C5 | 0.25* | -0.04 | † | -0.43* | 0.04 | † |  | 0.17 | -0.31* |  | -0.14 | -0.00 |
| C6 | -0.14 | -0.27* |  | 0.10 | 0.16 |  |  | -0.33* | 0.21 |  | -0.03 | -0.08 |
| C7 | 0.09 | 0.13 |  | -0.04 | -0.11 |  |  | 0.18 | -0.12 |  | 0.04 | 0.02 |
| C8 | 0.05 | -0.02 |  | -0.13 | 0.26* | † |  | 0.02 | 0.10 |  | -0.07 | 0.19 |
| C9 | -0.34* | 0.47* | † | -0.13 | 0.06 |  |  | 0.10 | -0.05 |  | -0.38* | 0.43* |

* *p* < 0.05 Bonferroni-corrected (x 72) for correlation coefficient.

† *p* < 0.05 Fisher z- test for correlations between pain and control.

**Table S2. Subjective ratings for stimuli (***M* ± *SD***)**

|  | Categories of stimuli | | | |
| --- | --- | --- | --- | --- |
| Ratings | affective control | physical control | affective pain | physical pain |
| Pain intensity | 5.09 ± 7.34 | 8.49 ± 9.68 | 53.64 ± 21.43 | 71.43 ± 17.18 |
| Arousal | 16.79 ± 18.72 | 22.23 ± 18.74 | 54.48 ± 20.13 | 72.57 ± 17.53 |
